# MAGE-C2/CT10 Protein Expression Is an Independent Predictor of Recurrence in Prostate Cancer

**DOI:** 10.1371/journal.pone.0021366

**Published:** 2011-07-06

**Authors:** Lotta von Boehmer, Lukas Keller, Ashkan Mortezavi, Maurizio Provenzano, Giovanni Sais, Thomas Hermanns, Tullio Sulser, Achim A. Jungbluth, Lloyd J. Old, Glen Kristiansen, Maries van den Broek, Holger Moch, Alexander Knuth, Peter J. Wild

**Affiliations:** 1 Clinic of Oncology, University Hospital Zurich, Zurich, Switzerland; 2 Clinic of Urology, University Hospital Zurich, Zurich, Switzerland; 3 Institute of Surgical Pathology, University Hospital Zurich, Zurich, Switzerland; 4 Ludwig Institute for Cancer Research, New York Branch, Memorial Sloan-Kettering Cancer Center, New York, New York, United States of America; University of Chicago, United States of America

## Abstract

The cancer-testis (CT) family of antigens is expressed in a variety of malignant neoplasms. In most cases, no CT antigen is found in normal tissues, except in testis, making them ideal targets for cancer immunotherapy. A comprehensive analysis of CT antigen expression has not yet been reported in prostate cancer. MAGE-C2/CT-10 is a novel CT antigen. The objective of this study was to analyze extent and prognostic significance of MAGE-C2/CT10 protein expression in prostate cancer. 348 prostate carcinomas from consecutive radical prostatectomies, 29 castration-refractory prostate cancer, 46 metastases, and 45 benign hyperplasias were immunohistochemically analyzed for MAGE-C2/CT10 expression using tissue microarrays. Nuclear MAGE-C2/CT10 expression was identified in only 3.3% primary prostate carcinomas. MAGE-C2/CT10 protein expression was significantly more frequent in metastatic (16.3% positivity) and castration-resistant prostate cancer (17% positivity; p<0.001). Nuclear MAGE-C2/CT10 expression was identified as predictor of biochemical recurrence after radical prostatectomy (p = 0.015), which was independent of preoperative PSA, Gleason score, tumor stage, and surgical margin status in multivariate analysis (p<0.05). MAGE-C2/CT10 expression in prostate cancer correlates with the degree of malignancy and indicates a higher risk for biochemical recurrence after radical prostatectomy. Further, the results suggest MAGE-C2/CT10 as a potential target for adjuvant and palliative immunotherapy in patients with prostate cancer.

## Introduction

When prostate cancer is localized in the prostate, the treatment of choice is prostatectomy or irradiation. However, when the tumor relapses or is already metastatic at diagnosis, therapy is problematic. Castration has been the main treatment option for unconfined disease for more than 50 years. However, patients frequently progress after endocrine treatment [1]. Occurrence of castration resistance is associated with poor prognosis and only palliative therapy is available in such advanced tumor stages. Immunotherapy targeting cancer-testis (CT) antigens are promising new treatment modalities for advanced lung cancer, ovarian cancer and melanoma patients [2],[3],[4],[5],[6],[7],[8],[9] but CT antigens, except NY-ESO-1 [10], haven't been employed as vaccine targets for prostate cancer.

In most cases, CT antigens are only expressed in germ cells of the human testis. To date, more than 100 CT antigens have been identified, which belong to at least 44 distinct families. CT antigens mapping to chromosome X are referred to as CT-X antigens and distinguished from Non-X CT antigens located on other chromosomes [11]. The expression of CT-X antigens varies greatly between different tumor types and are more prevalent in higher grade and advanced stage tumors [12],[13],[14],[15]. They are most frequently expressed in melanomas [16], bladder [17],[18], lung [12], ovarian [19], and hepatocellular carcinomas [20], and are uncommon in renal cell carcinoma [21], colon cancer [22], and hematological malignancies [23].

Interestingly, a member of the CT antigens, *MAGE-A11* appears to directly contribute to the development of androgen independent prostate tumor growth by stimulating the activity of the androgen receptor [24]. *MAGE-A11* expression is regulated by DNA methylation status: in castration-recurrent prostate tumors, *MAGE-A11* is upregulated, correlating with hypomethylation of discrete CpG sites adjacent to the transcriptional start site of the gene, by contrast, the methylation status of other regions of the *MAGE-A11* promoter CpG island does not correlate with gene expression [24]. Furthermore, treatment of prostate cells with decitabine causes upregulation of *MAGE-A11* expression [24],[25].

Studies analysing mRNA expression [26],[27],[28],[29],[30] and immunohistochemical analyses [31],[32],[33] of several CT antigens have been performed. There is evolving evidence that NY-ESO-1 expression in the tumor changes from negative to positive during the progression of disease [34]. In prostate cancer, which is known to be a relatively slow progressing disease, expression ranges stage dependant from 5% to 30% [29],[31]. Recently, an antibody against MAGE-C2/CT-10, a novel CT antigen has been generated [35]. The MAGE-C2/CT-10 gene shows significant homology with the MAGE-C1/CT-7 gene and both genes map in close proximity to chromosome Xq27.13. MAGE-C2/CT-10 was originally identified in a melanoma cell line. Until now, MAGE-C2/CT10 mRNA expression in prostate cancer was analysed in few prostate samples, only: In a study by Prikler et al., 12 castration-resistent and eight hormone sensitive tumors were CT10 negative. Furthermore, Lucas et al. found one of ten prostate cancer tissues to be CT10 positive [28],[30].

We have reported MAGE-C2/CT-10 in a large proportion of hepatocellular carcinoma (HCC) [20]. While testing the MAGE-C2/CT-10 antibody in a multi-tumor tissue microarray, we identified MAGE-C2/CT10 protein expression in single PCa cases. Based on this observation, we performed a comprehensive tissue microarray-based analysis of PCa. We demonstrate that protein expression of MAGE-C2/CT10 is found in a substantial subset of prostate cancer, mainly metastatic and castration resistant primary tumors. We further show that MAGE-C2/CT10 expression was identified as predictor of biochemical recurrence after radical prostatectomy, independent of known risk factors.

## Materials and Methods

### Prostate tissue microarrays

A total of 468 formalin-fixed, paraffin-embedded prostate tissues was retrieved from the archives of the Institute of Surgical Pathology, University Zurich, Switzerland and a tissue microarray (TMA) was constructed as described previously [36]. The TMA included a series of 348 consecutive (non-selected) radical prostatectomy specimens with prostate cancer, 29 castration resistent prostate cancer samples, 18 lymph node metastases, 28 distant metastases (bone, lung, urinary bladder) and 45 benign prostatic hyperplasia samples. H&E-stained slides of all specimens were re-evaluated by experienced pathologists (P.J.W., H.M.) to identify representative areas for TMA construction. Tumor stage and Gleason score of the cohort were assigned according to the International Union Against Cancer (UICC) and WHO/ISUP criteria [37]. Median follow-up of the cohort was 71 months (0-163). The raw data of the tissue microarray have been deposited under (link will be sent). The Zurich cantonal scientific ethics committee for pathology (KEK) approved the study and waived the need for consent (Ref. No. StV-Nr-05/2007).

### Immunohistochemistry

The expression of MAGE-C2/CT10 was analyzed immunohistochemically as reported recently [20]. Clinical and pathological parameters of the prostate cancer cases included in the TMA are summarized in Table S1. Consecutive 3 µm sections were cut from TMA blocks and mounted on glass slides (Super-Frost Plus, Menzel, Braunschweig, Germany). For immunohistochemical staining the Ventana Benchmark automated staining system (Ventana Medical Systems, Tucson, AZ) and Ventana reagents were used. After deparaffinization in xylene, slides were rehydrated in decreasing concentrations of ethanol. Endogenous peroxidase was blocked using Ventana endogenous peroxidase blocking kit after a rinse with distilled water. For antigen retrieval slides were heated with cell conditioning solution (CC1, Ventana) according to manufacturer's instructions. For the detection of MAGE-C2/CT-10, the mAb CT10#5 previously generated by our group [35], for the detection of MAGE-C1/CT7, the clone CT7-33, DAKO A/S and for NY-ESO-1 the clone E978, ZYMED were employed and adjusted to the Ventana Benchmark system after performing titrations (optimal dilution 1∶100; 1∶80, 1∶50 respectively). iVIEW-DAB was used as chromogen. Normal testicular tissue was chosen as internal positive control for MAGE-C2/CT10, MAGE-C1/CT7 and NY-ESO-1 expression. MAGE-C2/CT10 expression was nuclear, MAGE-C1/CT7 and NY-ESO-1 expression was cytoplasmic and nuclear. For negative controls, the primary antibody was omitted. Two investigators (L.v.B., L.K.) performed a blinded evaluation of the slides for MAGE-C2/CT10, MAGE-C1/CT7 and NY-ESO-1 expression. Non-interpretable results, due to lack of carcinoma tissue, presence of necrosis or crush artifact, were excluded from the analysis. At least 100 cells were counted in each TMA core. Nuclear MAGE-C2/CT10 immunoreactivity was evaluated using a semi-quantitative, stepwise scoring system: negative (0% of cell nuclei stained); weak nuclear staining (1–10% of nuclei stained); moderate nuclear staining (11–50% of nuclei stained); strong nuclear staining (51 to 100% of nuclei stained). Searching for cutoffs in an unbiased way is a major problem in immunohistochemical studies dealing with a continuous readout. The median nuclear CT10 immunoreactivity in prostatectomy cases (median 0%) was chosen as cutoff. Accordingly, positive nuclear CT10 immunoreactivity was defined as nuclear staining in at least 1% of target cells. NY-ESO-1- and CT7- staining is cytoplasmic or nuclear, immunoreactivity was evaluated using a semi-quantitative, stepwise scoring system: negative (0% of cells stained); weak nuclear staining (1–10% of cells stained); moderate staining (11–50% of cells stained); strong staining (51 to 100% of cells stained). The median NY-ESO-1 and CT7 immunoreactivity (median 0%) was chosen as cutoff. Accordingly, positive staining for NY-ESO-1 and CT7 immunoreactivity was defined as nuclear or cytoplasmic staining in at least 1% of target cells.

### Statistical analyses of tissue microarray data

**Figure 1:**
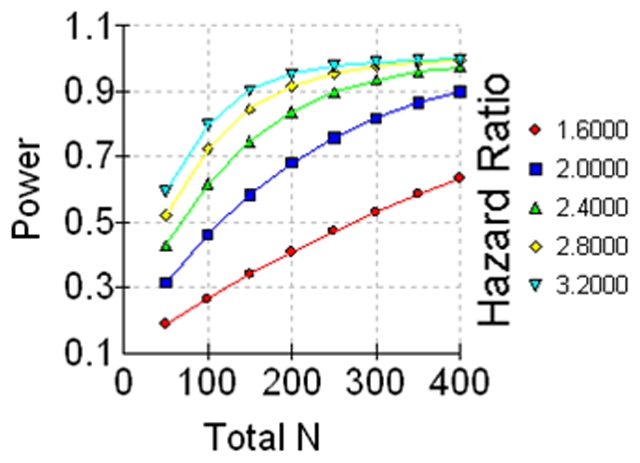
Estimation of statistical power *versus* total sample size N for different hazard ratios. MAGE-C2/CT10 expression could be observed in 11 of 341 (3.2%) prostatectomy specimens. The occurrence of MAGE-C2/CT10 expression should double the risk of PSA recurrence during follow up, resulting in a hazard ratio of approximately 2.0. Accordingly, the available sample size of 341 analyzable patients would be sufficient to detect a difference concerning PSA recurrence with a significance of p<0.05 and a power of approximately 86%. For higher hazard ratios (2.4, 2.8, 3.2) a statistical power of approximately 99% was calculated.

SPSS version 17.0 (SPSS, Chicago, IL, USA) was used for statistical analyses. P-values<0.05 were considered significant. In case of multiple tests the Bonferroni-Holm procedure was applied. Contingency table analysis and two-sided Fisher's exact tests were used to study statistical associations between clinicopathological and immunohistochemical data. For the comparison of two independent samples the non-parametric Mann-Whitney U-test was calculated. Time to PSA recurrence (cut off≥0.1 ng/ml) was selected as clinical end point. Recurrence-free survival (RFS) curves were calculated by the Kaplan-Meier method with significance evaluated by two-sided log-rank statistics. Patients were censored at the time of their last tumor-free clinical follow-up visit. Patients not reaching PSA nadir (<0.1 ng/ml) postoperatively were excluded. A stepwise multivariable Cox regression model was adjusted, testing the independent prognostic relevance of MAGE-C2/CT10 immunoreactivity. The proportionality assumption for all variables was assessed with log-negative-log survival distribution functions. Statistical considerations regarding sample size are given in Figure 1. Calculations were performed using the respective models of the PASS 2008 software (NCSS, Kaysville, UT).

## Results

### MAGE-C2/CT10 expression in normal and malignant prostate tissue

**Figure 2:**
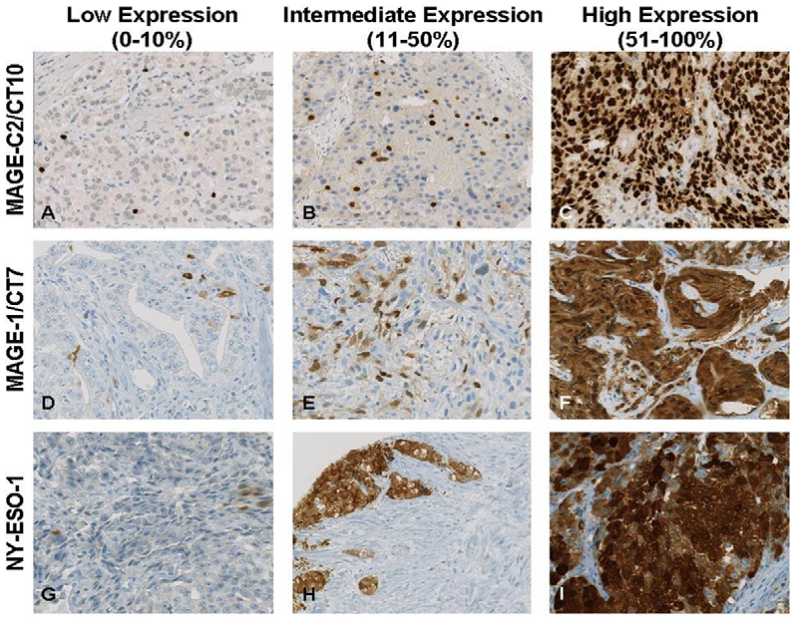
Representative immunohistochemical expression patterns from 1–10%, 11–50% and 51–100% of MAGE-C2/CT10 (A, B and C), MAGE-C1/CT7 (D, E and F) and NY-ESO-1 (G, H and I). A: Radical prostatectomy specimen (Gleason 4+3), B: Bone metastasis of prostate cancer, C: Bone metastasis of prostate cancer, D: Radical prostatectomy specimen (Gleason 4+3), E: Castration-resistant prostate cancer (Gleason 5+5), F: Palliative transurethral resection of prostate cancer (Gleason 4+5), G: Bone metastasis of prostate cancer, H: Palliative transurethral resection of prostate cancer (Gleason 5+4), I: Castration-resistant prostate cancer (Gleason 5+4). Original magnification: 200×; Magnification bar: 20 µM.

**Figure 3:**
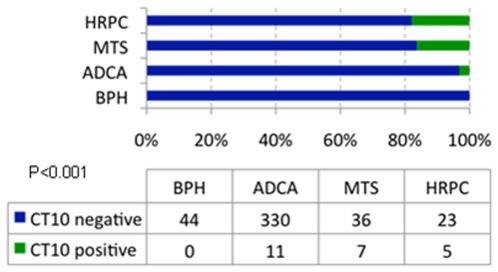
Cumulative bar chart representing nuclear immunoreactivity for MAGE-C2/CT10 in different prostate tissue types, showing increased expression from prostatic hyperplasia to organ-confined prostate cancer to metastatic and castration resistant disease (p<0.001). BPH: benign prostatic hyperplasia; ADCA: organ-confined adenocarcinoma of the prostate; MTS: prostate cancer metastasis; CRPC: castration-resistant prostate cancer.

**Figure 4:**
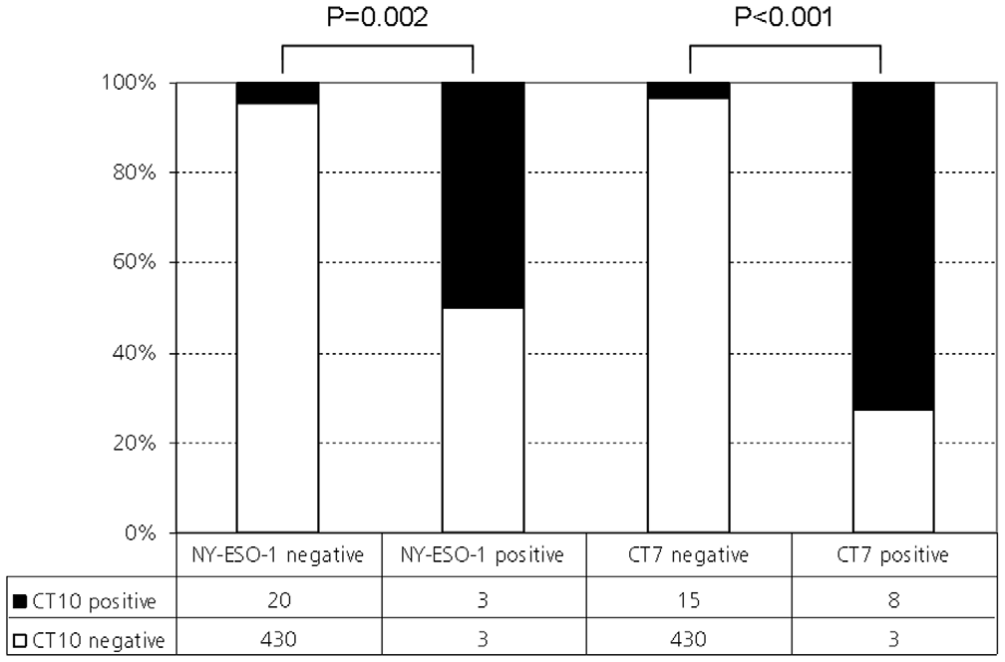
Significant coexpression of MAGE-C2/CT10 with NY-ESO-1 and MAGE-C1/CT7 on the tissue microarray.

Comprehensive clinical and histopathologic data are given in Table S1. In total, 456 of 468 cores (97.4%) could be evaluated for MAGE-C2/CT10 immunoreactivity. A representative MAGE-C2/CT10 staining pattern is shown in Figure 2A–C. MAGE-C2/CT10 expression was not detected in prostatic hyperplasia. Organ-confined cancers showed nuclear MAGE-C2/CT10 expression in 3.3% (11/330) cases, whereas metastatic and castration resistant disease were positive in 16.3% (7/36) and 17% (5/23) of cases, respectively. Nuclear MAGE-C2/CT10 staining progressively increased from prostatic hyperplasia to prostate-confined cancer to metastatic and castration resistant disease (Figure 3; p<0.001). As shown in Figure S1, differential CT10 expression between normal and neoplastic tissue could be observed: the percentage of CT10 positivity significantly increased from benign prostatic hyperplasia to organ confined prostate cancer to castration resistant prostate and metastatic disease, including lymph node and bone metastases. As previously described [1], neuroendocrine differentiation is more prevalent in castration resistant prostate cancer. However, no coexpression of CT10 and neuroendocrine markers such as synaptophysin and chromogranin could be detected (Figure S2). No correlation between nuclear MAGE-C2/CT10 expression and age at diagnosis, Gleason score, tumor stage, nodal status, surgical margin status or preoperative PSA levels was found (Table 1). Interestingly, CT10 was significantly co-expressed with other CT antigens (Fig. 4), like NY-ESO-1 and CT7 (Fig. 2D–I).

**Table 1:** Clinicopathological characteristics in relation to CT10 immunoreactivity in radical prostatectomy tumor specimens.

Variable	Characteristics	CT10 immunoreactivity		
		negative	positive	p
Age at diagnosis (grouped)	<64 years	143	4	1.000^a^
	≥ 64 years	178	6	
Gleason score (grouped)	5–6	55	0	0.152^b^
	7	193	6	
	8–10	80	5	
Tumor stage (grouped)	pT2a-c	209	6	0.558^b^
	pT3a-b	105	5	
	pT4	13	0	
Nodal status	pN0	254	10	1.000^a^
	pN1	17	0	
Surgical margin status	negative	212	6	0.524^a^
	positive	111	5	
Preoperative PSA levels	<10 ng/mL	131	3	0.520^a^
	≥ 10 ng/mL	157	7	

a Fisher's exact test, two-sided; bold face representing p<0.05.

b Pearson Chi-Square, Asymp. Sig., two sided.

p = p-value.

### MAGE-C2/CT10 and prognosis

**Figure 5:**
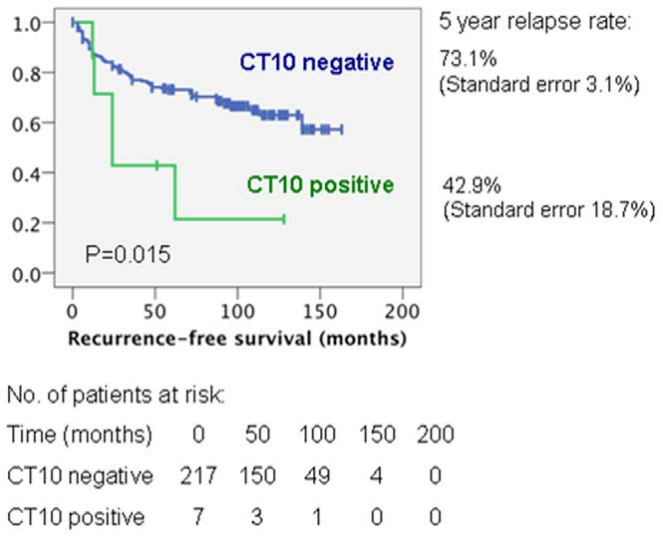
Kaplan–Meyer curves regarding disease-free survival of patients with no MAGE-C2/CT10 expression (blue line) *vs.* patients with positive MAGE-C2/CT10 expression (green line) in invasive prostate carcinomas receiving radical prostatectomy.

Patients with MAGE-C2/CT10 positive prostate cancers were compared with negative cases regarding RFS by univariate Cox regression analysis (Table 2). MAGE-C2/CT10 expression was significantly associated with shorter RFS (p = 0.015; Figure 5). Patients with MAGE-C2/CT10 positive tumors had a median RFS of 51 months (95% confidence interval 17-86 months) compared to 116 months (95% confidence interval 107–125 months) for patients with MAGE-C2/CT10 negative tumors. Besides the expression of MAGE-C2/CT10, increased Gleason score (p<0.001), tumor stage (p<0.001), surgical margins (p<0.001) and preoperative PSA level (p<0.001) were significantly associated with shorter RFS time.

**Table 2:** Univariate Cox regression analysis.

Variable	Characteristics	Recurrence-free survival		
		HR	95% CI	p*
Age at diagnosis		1.006	0.964–1.050	0.772
Gleason score (grouped)	5–6 vs 7 *vs* 8–10	2.675	1.831–3.906	<0.001
Tumors stage (grouped)	pT2a–c *vs* pT3a-4	2.336	1.625–3.356	<0.001
Surgical margin status	negative *vs* positive	3.268	2.069–5.161	<0.001
Preoperative PSA level	<10 ng/mL *vs*≥10 ng/mL	2.317	1.432–3.748	0.001
CT10 immunoreactivity	negative *vs* positive	2.913	1.170–7.252	0.022

*P-values<0.05 are marked in bold.

HR = Hazard ratio.

In a multivariate analysis, a Cox regression model was developed for assessment of the RFS rate. Characteristics of variables are shown in Table 3. Only MAGE-C2/CT10 expression, Gleason score, tumor stage, surgical margin status and preoperative PSA levels were considered. All variables, including MAGE-C2/CT10 expression (p = 0.03), remained significant. The hazard ratio for MAGE-C2/CT10 expression was 2.770 (95% confidence interval 1.106–6.934).

**Table 3:** Multivariate Cox regression analysis.

Variable	Characteristics	Recurrence-free survival		
		HR	95% CI	p*
Gleason score (grouped)	5–7 *vs* 8–10	2.237	1.314–3.807	0.003
Tumors stage (grouped)	pT2a-c *vs* pT3a-pT4	1.950	1.184–3.212	0.009
Surgical margin status	negative *vs* positive	2.598	1.599–4.223	<0.001
Preoperative PSA level	<10 ng/mL *vs*≥10 ng/mL	1.921	1.135–3.252	0.015
CT10 immunoreactivity	negative *vs* positive	2.770	1.106–6.934	0.030

*p-values<0.05 are marked in bold.

HR = Hazard ratio.

## Discussion

In the present study, we analyzed the presence of MAGE-C2/CT10 protein in a representative cohort of patients with prostate cancer and found MAGE-C2/CT10 to be frequently expressed in advanced prostate cancer; i.e. in metastatic and castration-resistant disease. Moreover, we identified nuclear MAGE-C2/CT10 expression as a predictor of biochemical recurrence after radical prostatectomy, which was independent of the well established predictive factors including preoperative PSA, Gleason score, tumor stage, and surgical margin status.

We detected nuclear MAGE-C2/CT10 positivity in 16.3% and 17% of patients with metastatic and castration resistant disease respectively, but only in 3.3% of organ confined PCa. This finding is of clinical significance, because a subgroup of patients with advanced, castration resistant PCa may be considered for immunotherapy in the future. Previous studies have shown that MAGE-C2/CT10 is able to induce specific immune responses in the autologous host. Cytotoxic T lymphocytes directed against MAGE/CT-10 epitopes have been found in melanoma patients and antibodies directed against MAGE-C2/CT-10 were detected in melanoma and patients [38],[39],[40],[12],[41].

MAGE-C2/CT10 belongs to the MAGE-family of CT antigens [42]. In a recent study, Yang et al. demonstrated that MAGE-C2 can act as co-repressor of p53 by binding to KAP1 enhancing suppression of p53. These results suggest that MAGE-C2 contributes to the development of malignancies by providing a survival advantage [43]. MAGE gene expression is epigenetically repressed by promoter region methylation in most cells but factors controlling MAGE gene promoter methylation have not been fully identified. Yang et al. have shown that MAGE gene expression is epigenetically controlled by the KIT tyrosine kinase [44]. Understanding the factors controlling MAGE gene expression may allow more effective therapeutic strategies targeting MAGE antigens [45],[46],[47].

While our study corroborates previous studies reporting low incidence of CT antigens in organ-confined prostate cancer, the increased MAGE-C2/CT10 antigen expression in advanced PCa was an unexpected novel finding. Also CT antigen expression has not yet been analyzed in a larger cohort of PCa patients. To our knowledge, only 30 tumors have been analyzed for MAGE-C2/CT10 mRNA expression previously: positivity was reported in 1/10 and 0/20 tumor samples [30],[32]. In other tumors, MAGE-C2/CT10 protein was previously identified in 34%-48% of hepatocellular carcinomas [20],[48], in 43% of multiple myeloma [49], in 20% of high-grade urothelial carcinomas of the urinary bladder [18], in 20% of head and neck cancers [28], and in 43% of melanomas, respectively. The reported 5% prevalence of MAGE-C2/CT-10 expression in colorectal cancers [50] is comparable to our finding of rare MAGE-C2/CT-10 expression in primary PCa. A poor survival was observed in advanced MAGE-C2/CT-10-positive urothelial carcinoma of the urinary bladder, but MAGE-C2/CT-10 expression had no prognostic impact in HCC.

Importantly, our study identified MAGE-C2/CT-10 as an independent predictor of biochemical recurrence after radical prostatectomy, providing a potential basis for better prognostication and treatment stratification of patients with PCa. The widespread use of serum prostate-specific antigen (PSA) screening has led to the identification of an increasing number of asymptomatic low-stage tumors in younger men [51],[52]. A yet unanswered important clinical question is if those patients require treatment and if so, how aggressively should this potential treatment be. Patients with localized disease are preferentially being treated with either radical prostatectomy or radiation therapy, both with curative intent [53],[54]. Currently, prognostication and treatment stratification at the time of diagnosis are based on clinical stage, biopsy Gleason grade, and serum PSA levels. In cases treated by radical prostatectomy, prognosis can be refined by using pathological stage and Gleason grade. However, these prognostic indicators do not accurately predict clinical outcome for individual patients. Improved markers are needed to determine which patients are at risk and should therefore be treated more aggressively. MAGE-C2/CT-10 should be added to the list of proposed prognostic tumor progression markers, including MUC1 [55], AZGP1 [55], EZH2 [56], E2F3 [57], Ki67 [58],[59], and CD10 [60].

In conclusion, our data provide evidence that MAGE-C2/CT-10 may be a candidate for adjuvant and palliative vaccination in a subset of patients with advanced prostate cancer. In addition, MAGE-C2/CT-10 expression in early tumor stages indicates a higher risk for biochemical recurrence after radical prostatectomy.

## Supporting Information

Figure S1 Differential CT10 expression between normal and neoplastic tissue: the percentage of CT10 positivity per tissue microarray core significantly increased from benign prostatic hyperplasia to organ confined prostate cancer to castration resistent prostate and metastatic disease, including lymph node and bone metastases.
(TIF)

Figure S2 Whole sections of CT10 positive bone metastasis from two patients were stained for chromogranin (CRGA) and synaptophysin, two neuroendokrine markers. No coexpression of CT10 and neuroendocrine markers could be detected.
(TIF)

Table S1 Clinicopathological characteristics and results of immunohistochemistry for patients receiving radical prostatectomy.
(XLS)
