# Supplementary figures and images for: MAGE-C2/CT10 Protein Expression Is an Independent Predictor of Recurrence in Prostate Cancer
Source: PLoS One. 2011 Jul 6;6(7):e21366. doi: 10.1371/journal.pone.0021366 (PMC3130772; doi:10.1371/journal.pone.0021366)

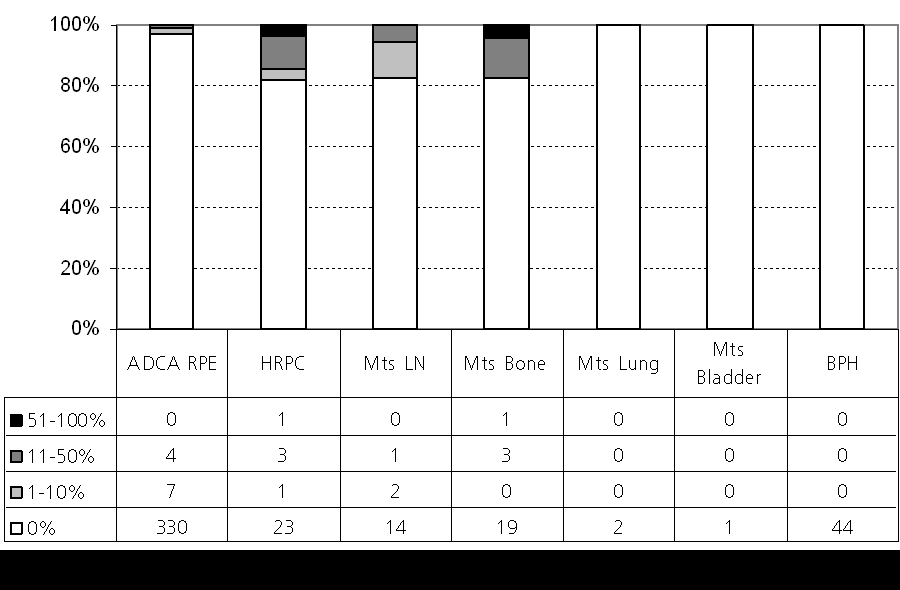

Supplement: Figure S1 — Differential CT10 expression between normal and neoplastic tissue: the percentage of CT10 positivity per tissue microarray core significantly increased from benign prostatic hyperplasia to organ confined prostate cancer to castration resistent prostate and metastatic disease, including lymph node and bone metastases. (TIF) [file pone.0021366.s001.tif]

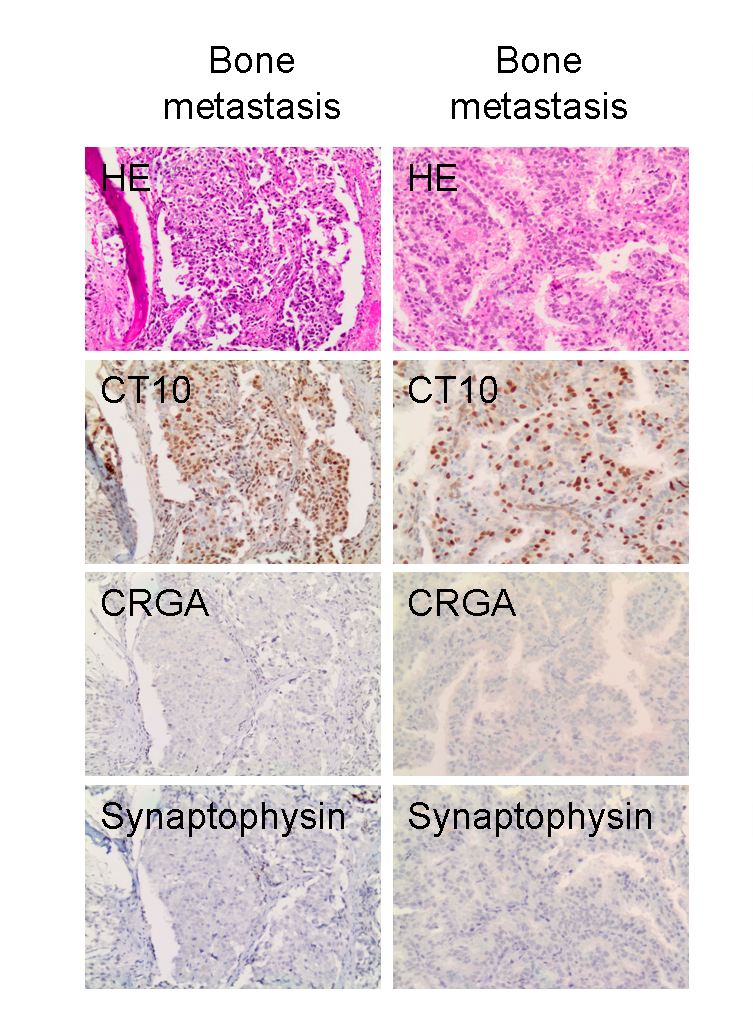

Supplement: Figure S2 — Whole sections of CT10 positive bone metastasis from two patients were stained for chromogranin (CRGA) and synaptophysin, two neuroendokrine markers. No coexpression of CT10 and neuroendocrine markers could be detected. (TIF) [file pone.0021366.s002.tif]
